# Adverse Reaction Following the Subarachnoid Injection of Xylazine in a Sheep

**DOI:** 10.3390/vetsci9090479

**Published:** 2022-09-05

**Authors:** Shaun Pratt, Sharon Jeong, Ben Ahern, Wendy Goodwin

**Affiliations:** School of Veterinary Science, Gatton Campus, The University of Queensland, Gatton, QLD 4343, Australia

**Keywords:** adverse reaction, subarachnoid injection, xylazine, sheep

## Abstract

**Simple Summary:**

To maintain high standards of veterinary care, it is crucial adverse events are reported. This case report describes the adverse cardiovascular and respiratory responses to xylazine—a veterinary sedative and pain relief drug. While adverse effects are known to occur following vascular injection of xylazine during anaesthesia in sheep, this is the first report to detail the adverse responses following the injection of xylazine into the subarachnoid space. Injecting xylazine into the subarachnoid space (the space around the spinal cord containing spinal fluid) is commonly performed for the management of pain in sheep; as most clinicians believe this method carries significantly less risks for the patient than vascular routes of administration. The profound cardiovascular and respiratory depression detailed here is therefore of interest to veterinary professionals and scientists involved in the anaesthesia and/or pain management of sheep.

**Abstract:**

Alpha_2_ receptor agonists are frequently used to provide sedation and analgesia in sheep. There are numerous reports of adverse pulmonary effects following intravenous (IV) injection; however, adverse effects following subarachnoid injection (SAI) are underreported. An adult Merino wether was one of eighteen animals anaesthetised during an experimental trial modelling intervertebral disc injury. The animal was premedicated with methadone 0.1 mg/kg and midazolam 0.3 mg/kg IV. Anaesthesia was induced using alfaxalone IV and it was maintained using isoflurane, delivered in 100% oxygen by controlled mechanical ventilation. An SAI of xylazine 0.05 mg/kg diluted to 1 mL with 0.9% saline was performed at the lumbosacral site prior to recovery. This resulted in rapid narcosis, oxygen dependency and ventilatory compromise. Treatment with frusemide 1 mg/kg IV and salbutamol 0.2 mg inhaled did not attenuate the adverse cardiopulmonary effects. A rapid improvement in all physiological variables was seen following high dose atipamezole 0.05 mg/kg IV. This case report adds to the current knowledge regarding the risk for potential side effects when using alpha_2_ receptor agonists, such as xylazine, for the sedation or regional analgesia in sheep.

## 1. Introduction

Alpha_2_ receptor agonists (alpha_2_-agonists) are frequently used to provide sedation and analgesia in sheep. Numerous reports detail the cardiorespiratory effects of commercially available alpha_2_-agoinsts, with the most profound responses reported to occur after intravenous (IV) injections of xylazine during general anaesthesia [1,2,3]. Interestingly, there are few reports of adverse effects following subarachnoid injections of xylazine, with many studies describing comparably mild cardiorespiratory depression in sheep [4] and goats [5]. This has resulted in the widespread use of xylazine administered via subarachnoid injection for the management of orthopaedic pain in small ruminants [4]. In contrast, this case report describes the profound cardiorespiratory responses and neuro-depression following the subarachnoid injection of xylazine in a sheep, and the subsequent clinical improvement after its antagonism with high dose atipamezole IV. The sheep was one of eighteen animals anaesthetised during an experimental trial (2020/AE000365) which modelled intervertebral disc injury using a previously described technique [6].

## 2. Report

A middle aged, castrated male Merino sheep weighing 47.2 kg was premedicated with IV methadone (Methadone; Ilium-Troy, Glendenning, NSW, Australia) 0.2 mg/kg and midazolam (Midazolam; Pfizer, Mulgrave, VIC, Australia) 0.4 mg/kg. The animal was pre-oxygenated with a face mask prior to induction of anaesthesia with IV alfaxalone (Alfaxan; Jurox, Rutherford, NSW, Australia) 2.1 mg/kg administered over approximately 90 s. After 20 mg lidocaine (Lignocaine20; Ilium-Troy) was deposited topically onto the arytenoid cartilages, orotracheal intubation was performed with an 11 mm internal diameter cuffed endotracheal tube, which was connected to the foal circuit of a Mallard large animal circle system (Mallard Medical, Redding, CA, USA). An oro-ruminal tube was placed and the sheep was positioned in right lateral recumbency. Anaesthesia was maintained using isoflurane (Isofol; Zoetis, Sydney, NSW, Australia) in oxygen, to a depth adequate for surgical manipulation (end expiratory isoflurane concentration 1.4–1.8%). Physiological variables were monitored using a multiparameter monitor (Bionet BM7Vet; Bionetus, Tustin, CA, USA) and included: pulse oximetry, capnography, electrocardiography, oscillometric arterial blood pressure, inspired and expired gas monitoring and nasopharyngeal temperature. Controlled mechanical ventilation (CMV) was set to deliver a peak inspiratory pressure (PIP) of 20 cm H_2_O and an inspiratory time of 1.2 s. Respiratory rate (*f*_R_) was adjusted to maintain end expiratory normocapnia. Hartmann’s solution was provided via a gravity giving set at 5 mL/kg/hr. Additional treatments included ceftiofur (Accent; Zamira Life Sciences, South Yarra, VIC, Australia) 5 mg/kg IV at draping, methadone 5 mg IV at surgical incision, meloxicam (Meloxicam20; Ilium-Troy) 1 mg/kg subcutaneous at surgical completion and two fentanyl 75 μg/h transdermal patches (Fentanyl; Sandoz, Macquarie Park, NSW, Australia) applied to the left antebrachium at surgical completion.

Anaesthesia was maintained without complication for the 35 min surgical period. Following the completion of surgery (72 min postinduction of anaesthesia), a subarachnoid injection (SAI) of xylazine (Xylazil20; Ilium-Troy) 0.05 mg/kg diluted to a total volume of 1 mL with sterile 0.9% saline was administered at the lumbosacral site. A new, unbreeched xylazine multidose bottle was used. The calculated dose of xylazine was drawn into a 0.3 mL insulin syringe (BD; Becton Dickinson Company, Macquarie Park, NSW, Australia) and added to a 3 mL syringe (BD; Becton Dickinson Company) containing the appropriate volume of 0.9% saline. The diluted xylazine solution was mixed by inverting the syringe several times.

The injection technique was extrapolated from Hall et al. [1]. Briefly, the sheep was positioned in sternal recumbency with both pelvic limbs pulled cranially. The lumbosacral junction was identified on palpation of the adjacent anatomical landmarks [1]. A 21-gauge, 75 mm Quincke-type spinal needle (BD; Becton Dickinson Company) with the bevel orientated cranially, was slowly advanced through the skin, the supraspinous ligament and the interarcuate ligament until a pop was appreciated. The visual return of cerebral spinal fluid and the absence of blood contamination upon aspiration confirmed correct needle placement. The injectate was administered slowly over 10 s without resistance.

The physiological variables and clinical observations/signs of anaesthetic depth immediately prior to the SAI, following the SAI at 0 and 3 min, and following major interventions are shown in Table 1. Almost immediately following completion of the SAI, the sheep became bradycardic (heart rate 50 beats/min), end expiratory carbon dioxide concentration decreased (P_E_CO_2_ 30 mm Hg) and it became tachypnoeic, with rapid and forceful respiratory efforts over the CMV settings. The palpebral reflex, jaw tone and general muscle tone reduced rapidly.

The sheep was maintained in sternal recumbency while the inhalant was turned off and the breathing system disconnected and purged with oxygen using the emergency flush valve. While disconnected (approximately 3 min) the sheep became hypoxemic on room air as evidenced by an SpO_2_ of 60%. Cyanosis of the mucous membranes was also noted. Oxygenation partly improved to an SpO_2_ of 91% following reconnection to the breathing system and provision of 100% inspired oxygen concentration (F_I_O_2_). During this time, peripheral pulse quality diminished and measurement of non-invasive arterial blood pressure failed. The sheep’s anaesthetic depth deepened, as indicated by a diminished jaw tone and negative palpebral reflex. Ventilation was laboured and an audible wheeze was appreciable. The oropharynx was assessed for signs of ruminal contents using a laryngoscope and after none were observed, the endotracheal tube was removed to check for a possible obstruction. There was no evidence for an obstruction within the lumen of the removed tube and the ventilation and physiological parameters remained unchanged following re-intubation. Salbutamol (Ventolin Reliever; Allen & Hanburys, London, UK) 0.2 mg was administered via the gas sampling port of the rebreathing circuit during inspiration of CMV and frusemide (Frusemide; Ilium-Troy) 1 mg/kg was administered IV.

At 30 min after the SAI and following the described interventions, the animal’s condition remained relatively unchanged (Table 1). The sheep remained bradycardic, hypotensive (mean arterial blood pressure < 60 mm Hg), tachypneic and deeply anaesthetised (negative palpebral reflex and no jaw tone). At 40 min after the SAI, high dose atipamezole (Atipamezole; Ilium-Troy) 0.05 mg/kg was administered IV as a bolus over 10 s. Almost immediately the sheep regained muscle tone and its palpebral reflex returned. A coinciding improvement in physiological variables was also noted. As the animal began to regain consciousness approximately 3 min later, alfaxalone 20 mg IV was administered slowly. Ten minutes later, adequate oxygenation was assessed on room air (F_I_O_2_ 21%) during spontaneous ventilation, before the animal was moved to a padded recovery stall and placed in sternal recumbency. Methadone 2.5 mg IV was administered once in recovery and SpO_2_ monitoring continued until the animal was extubated when chewing, 55 min after the SAI.

No re-narcosis was observed during the recovery period following extubation and the sheep stood at 220 min after the SAI. Buprenorphine (Temvet, Ilium-Troy) 0.01 mg/kg intramuscular was administered twice for additional analgesia at 6 hourly intervals from the last methadone dose. Unilateral left pelvic limb weakness, ataxia and mild proprioceptive deficits (knuckling and limb interference) were noted once the animal was standing. Despite this, the sheep was observed eating and demonstrating normal social behaviour with the four other sheep in its enclosure. The proprioceptive deficits persisted and a hindlimb dorsal splint was placed and changed every other day until full neurological function returned to the left pelvic limb 2 weeks post-surgery.

## 3. Discussion

The activation of centrally located alpha_2_ receptors reduce sympathetic outflow from the locus coeruleus and promote parasympathetic activity within the rostral ventrolateral medulla [7]. This produces the hypnosis, sedation and cardiovascular (sustained hypotension) and respiratory (hypoventilation) depression characteristic of alpha_2_-agonists in most species. In comparison, the adverse responses to systemic doses of xylazine in sheep; mainly hypoxemia, typically result from peripheral alpha_2_ receptor activation and indirect destruction to the pulmonary endothelium. Proposed mechanisms for this destruction include: the release of pro-inflammatory mediators from activated pulmonary intravascular macrophages, increases in hydrostatic pressure via vasospasm and dysregulated local coagulation [8]. This pathological complex occurs rapidly and is largely unresponsive to alpha_2_ receptor antagonists after 10 min [2]. The rapid clinical improvement following xylazine’s delayed (40 min) antagonism with high dose atipamezole, suggests major effusive pulmonary disease or advanced destruction to the pulmonary endothelium via peripheral mediation had not occurred. This is further supported by the lack of response to frusemide therapy.

Activation of peripherally located alpha_2_ receptors can increase airway pressure and previous reports have documented a mild reduction in airway resistance following atipamezole and atropine administration in sheep premedicated with xylazine [9]. However, the complete lack of response to salbutamol therapy suggests increases in airway resistance were not primarily responsible for the changes in respiratory rate, or pattern observed in this sheep. While silent regurgitation cannot be excluded, it seems unlikely that substantial bronchoconstriction and/or alveolar consolidation resulting from regurgitation would respond to atipamezole. Additionally, any perioperative aspiration would result in worsening clinical signs and would require prolonged postoperative management.

It is therefore hypothesised that the cardiorespiratory responses and neuro-depression observed in this sheep following the SAI of xylazine were centrally mediated. The resulting hypoxemia (as measured by pulse oximetry) likely occurred secondary to ineffective alveolar ventilation resulting from increases in central parasympathetic tone. This hypothesis is supported by the rapid clinical improvement following xylazine’s antagonism with high dose atipamezole IV and the theoretical re-initiation of interneural norepinephrine (sympathetic) conduction. The lack of arterial blood gas analysis and spinal cord histology, in addition to a small sample size, prevents definitive cause and effect analysis; however, plausible explanations for the atypical increases in central parasympathetic tone subsequent to the SAI of xylazine include: increased individual sensitivity to xylazine, direct intravascular injection, vascular absorption [10] or direct ependymal canal injection.

At a dose rate of 0.05 mg/kg, direct intravenous injection would likely result in peripheral alpha_2_ receptor activation and cause mild to moderate adverse pulmonary changes [2,8]. The severity of responses observed in this sheep, however, were uncharacteristically profound [4] and cannot be explained by peripheral alpha_2_ receptor activation alone. Furthermore, the rapid onset of cardiorespiratory depression suggests against absorption, as increases in plasma concentration following xylazine absorption would be slower to show an effect.

The injection of xylazine within the cerebral spinal fluid-filled ependymal canal is another possibility [11]. While the size and extension of the ependymal canal into the lumbosacral region in veterinary species is poorly described [11], a direct injection of xylazine into its lumen would result in the rapid delivery of highly concentrated xylazine to the forebrain and brain stem.

Finally, the persistence of localised neurological deficits to the left pelvic limb of the sheep may be explained by (a) local spinal inflammation resulting from the surgical technique, (b) spinal nerve laceration caused by the Quincke-type bevel of the spinal needle during the SAI, (c) intense local vasoconstriction resulting in spinal hypoxia and/or (d) perineural haemorrhaging from vascular trauma [12]. Poor patient positioning resulting in compressive neuropathy may also have contributed.

## 4. Conclusions

This report details the profound cardiorespiratory responses and neuro-depression following the subarachnoid injection of xylazine 0.05 mg/kg at the lumbosacral site in a sheep. The animal remained in a state of narcosis, oxygen-dependency and ventilatory compromise for approximately 40 min, despite therapy with CMV, salbutamol and frusemide. A clinical improvement was noted following high dose IV atipamezole 0.05 mg/kg administration, with the rapid return to consciousness and improvement in all cardiorespiratory variables. While a definitive cause remains unknown, a centrally mediated response appears most likely. This case report adds to the current knowledge regarding the risk for potential side effects when using alpha_2_ receptor agonists, such as xylazine, for the sedation or regional analgesia in sheep.

## Figures and Tables

**Table 1:** Cardiorespiratory variables for an adult castrated male Merino sheep undergoing experimental spinal surgery prior to and following the subarachnoid injection (SAI) of xylazine 0.05 mg/kg at the lumbosacral site, and its antagonism 40 min later with high dose atipamezole 0.05 mg/kg intravenous (IV).

	Immediately Prior to SAI	Immediately after SAI	3 min after SAI	30 min after SAI	Immediately after Atipamezole Injection	10 min after Atipamezole Injection
Heart rate (beats/min)	120	50	50	59	117	119
Pulse rate (pulse/min)	120	50	50	59	117	119
SpO_2_ (%)	97	95	60	91	95	94
FiO_2_ (%)	100	100	21	100	100	21
NIBP (mm Hg)	99/64 (76)	Unable to obtain a reading	92/50 (60)	77/44 (55)	120/70 (82)	128/67 (87)
P_E_CO_2_ (mm Hg)	48	30	28	30	40	40
Ventilation	CMV	CMV Tachypneic	CMV Tachypneic	CMV Tachypneic	CMV	Spontaneous Eupneic
Clinical observations and signs of anaesthetic depth	Positive palpebral reflex Modest jaw tone	Slight palpebral reflex Mild jaw tone	Negative palpebral reflex No jaw tone	Negative palpebral reflex No jaw tone	Positive palpebral reflex Modest jaw tone	Positive palpebral reflex Modest jaw tone

SpO_2_—saturation percentage of haemoglobin measured by pulse oximetry; F_I_O_2_—fraction of inspired oxygen concentration; NIBP—arterial blood pressure measured non-invasively via oscillometry presented as systolic/diastolic (mean); P_E_CO_2_—end expiratory carbon dioxide concentration measured by capnography; SAI—subarachnoid injection; CMV—controlled mechanical ventilation.

## Data Availability

Datasets presented in this study are available upon request from the corresponding author. The data is stored on The University of Queensland’s Research Data Manager and mediated access is available upon request.
